# Long-term follow-up of a low profile, coated, press-fit cup: the trabeculae oriented pattern (T.O.P.) acetabular system

**DOI:** 10.1007/s00590-021-02917-1

**Published:** 2021-03-03

**Authors:** L. Mosconi, L. Cavagnaro, A. Zanirato, E. Quarto, M. Lontaro Baracchini, M. Formica

**Affiliations:** 1 Clinica Ortopedica E Traumatologica, Ospedale Policlinico San Martino, Genova, Italy; 2 Ospedale Santa Corona, Pietra Ligure (SV), Italy

**Keywords:** Total hip arthroplasty, Tissue sparing surgery, Trabeculae oriented pattern, Coated acetabular cup

## Abstract

**Purpose:**

Total hip arthroplasties (THAs) are rising worldwide, as the functional request of patients who undergo this procedure. The trabeculae oriented pattern (TOP) is a modern cup, which follows the philosophy of the tissue sparing surgery (TSS). Focusing on clinical and radiological results and complications, the authors aim to highlight the outcomes of the TOP at a long-term follow-up (FU).

**Methods:**

A retrospective analysis was completed on THA performed with the TOP cup between 1997 and 2015. Five hundred and eighty-eight patients sustained surgery, for a total of 662 cup implanted. Four hundred and sixty patients (524 hips) were examined. Mean FU was 12 ± 4.9 years (range 5–22). Clinical (HHS, OHS and VAS) and radiological data were obtained. Every complication, reoperation or revision was recorded and analyzed.

**Results:**

Clinical evaluation revealed a HHS of 87.1 ± 13.8 an OHS of 41.3 ± 5.4, and a VAS of 1.2 ± 1.1. Acetabular osteolysis was observed in 53 hips. Overall survival rate of the cup was 90.5% (50 revisions), the main causes of cup substitution being aseptic loosening (AL) of the cup combined with the stem (26), of the cup only (13 cases) and periprosthetic joint infection (7 cases).

**Conclusion:**

TOP cup has demonstrated a good overall survivorship at a long-term FU, even compared with other coated cups, providing excellent clinical result with low rate of complications. Its association with a neck sparing stem permits a physiologic load transmission, reducing the stress shielding effect that could cause early implant mobilization.

## Introduction

Total hip arthroplasty (THA) has been described as the “operation of the century,” because of its excellent results in improving patients’ quality of life [1]: nowadays, uncemented components are the preferred choice for hip substitution, especially in younger patients.

Surgeons therefore must often measure themselves with patients’ high functional requests and expectations for a rapid recovery and return to preoperative activity levels. As well as stem components, also acetabular cups design evolved. Currently, the majority of acetabular cups relies on a press-fit mechanism for primary stability, with the possibility of adding cancellous screws. The bone-implant interface is made by osteoconductive or osteoinductive materials, such as porous or hydroxyapatite (HA) coating, porous tantalum or titanium. The trabeculae oriented pattern (TOP, Waldemar Link, Hamburg, Germany) cup was created following the philosophy of tissue sparing surgery (TSS) [2] and the idea that the prosthesis should reproduce native hip load transmission [3]. The concept of TSS has been introduced with the idea of substitute only pathologic tissue, in order to spare reliable bone facing the possibility of further revisions [2].

At our Institution, the first TOP cup, in combination with the collum femoris preserving (CFP, Waldemar Link, Hamburg, Germany) stem, was implanted in the late 90 s: the purpose of this paper is to report the long-term clinical and radiological outcomes as well as complications of TOP cup.

## Materials and methods

### Study design

A retrospective analysis was performed on a cohort of patients who underwent THA at our institution between 1998 and 2015. All patients provided their written and informed consent preoperatively to every medical and surgical procedures.

Inclusion criteria for patients’ selection were: THA with TOP cup, minimum follow-up (FU) of 5 years, complete clinical and radiological data. Patients with severe hip dysplasia (Crowe III or IV) or other severe joint deformities were excluded from case series. Demographic, clinical and surgical data were collected, including age at surgery and at final FU, sex, relevant comorbidities, smoking status, body mass index (BMI), preoperative diagnosis, surgical time, stem, liner, head features and cup dimension of all patients.

### TOP surgical philosophy

The TOP is a hemispherical cup made of Tilastan, a Ti 6Al 4Va alloy with a 160 μm porous surface coated with a 15-μm-thick calcium phosphate layer. It has a medial ventral recess, which diminishes the possibility of impingement between the cup and the collar stem, the iliopsoas tendon or the femoral nerve, tree teeth on the equator (similarly to those of treated cups) and tree holes for fixation screws, in order to improve primary stability. The liner is in ultra high molecular weight polyethylene (UHMWPE) or X-linked UHMWPE and presents a dissociation of 20° with the equator of the shell: the rationale is to improve the range of motion and permits the implantation of the cup even in dysplastic acetabulum, in which the inclination is often superior to the 55° recommended for shell positioning. The TOP presents 15 different sizes (from 40 to 68 mm of diameter, with a 2 mm increment): the inner diameter of the insert allows the use of ceramic or metal heads from 22 to 36 mm. A line-to-line reaming is recommended for correct cup implantation: trial cups and liners are available for intraoperative check.

### Surgical procedure

An accurate preoperative planning with component templates was developed, collegially debated, and a rescue option was prepared for every implant. Every surgery was performed through a modified Hardinge lateral approach, with the goal of minimizing muscle damage [4]. The aim of this approach is to minimize soft tissue damage and remove only pathologic tissue, in order to increase recovery and rehabilitation. A short-term antibiotic (ATB) prophylaxis was administered. Patients were mobilized with partial weight bearing using two crutches for the first week, following a rapid recovery of full ambulation: antithrombotic prophylaxis was carried out with low molecular weight heparin (LMWH) and compression socks for 45 days after the surgery.

### Clinical and radiological evaluation

Clinical evaluation was accomplished using the Harris Hip Score (HHS) to assess joint function from the clinician point of view, the Oxford Hip Score (OHS), patient-centered outcome, the visual analog scale (VAS) for assessment of groin or thigh pain [5]. HHS results were classified as excellent (HHS ≥ 90), good (89–80), fair (79–70) and poor (< 70), and OHS in satisfactory (40–48), mild (30–39), fair (20–29) and poor (0–19).

Every patient radiological evaluation was conducted on an anteroposterior (AP) plain pelvis X-ray with the legs in a slight (15°) internal rotation and a modified Dunn’ view [6]. The radiographs were analyzed by two experienced surgeons (MF and LF), blinded for clinical outcomes, for cup inclination (intended as the angle formed between the acetabulum and the transischial line [6]) and loosening, osteolysis according to DeLee and Charnley [7], Gruen’ zones for the stem [8], heterotopic ossifications (HO) according to Brooker classification [9], neck reabsorption ratio (NRR, only evaluated in case of neck sparing stems) [10] and leg length discrepancy (LDD) [11].

Every prosthesis-related complication, such as septic or aseptic loosening, dislocation, intra-/postoperative fractures, was recorded, as well as any reoperation (intended as any further surgery on the operated hip) or revision (any surgery that required fixed component exchange).

### Statistical analysis

Cup survival was reported as a Kaplan–Meier curve using GraphPad Prism 8, with cup revision as an end point. Continuous variables were expressed as mean ± standard deviation (SD), while categorical variables were reported as percentages or frequencies, using Microsoft Excel for Office 2019. For radiological parameters, Cohen’s kappa coefficient was used to assess correlation between the two testers, and it demonstrates a correlation of more than 90%.

## Results

Five hundred and eighty-eight patients underwent THA with the TOP cup between 1997 and 2014 at our institution: among them, 514 (87.4%) underwent unilateral hip substitution and 74 (12.6%) bilateral. Two hundred and eighty-nine (49.1%) were males and 299 (50.9%) females. Globally, 662 TOP cups were implanted during the examined period (374 on the right hip, 288 on the left one). Surgical diagnosis causing replacement was primary osteoarthritis (OA) in 541 patients (81.3%), femoral neck fracture in 60 (9.1%), femoral head necrosis in 33 (4.9%), post-traumatic OA in 14 (2.1%) and dysplasia in 13 (1.9%) epiphysiolysis in 1 (0.1%). Mean FU was 12 ± 4.9 years. At the last follow-up, 85 (14.4%) of patients were dead and 43 (7,3%) were lost, resulting in a total drop-out rate of 21,7% (128 patients), leaving 460 patients(524 hips) available for final statistical analysis. Patients sustained surgery at a mean age of 66 ± 15.5 years, and the mean age at the last FU was 75 ± 10.7 years. Regarding smoke status, 337 (73.3%) were non-smokers, 110 smokers (23.8%) and 13 (2.9%) former smokers: mean BMI was 26.5 ± 3.7. Mean surgical time was 89.1 ± 35.3 min. Cup size, liner and head features are resumed in Table 1; stem pairing in Table 2. Relevant comorbidities are summarized in Table 3.

**Table 1:** Cup dimension, liner and head features

	*N*	%
*Size*		
46	20	*2.9*
48	86	*12.9*
50	125	*18.8*
52	132	*19.9*
54	130	*19.5*
56	108	*16.3*
58	43	*6.5*
60	15	*2.2*
62	2	*0.4*
64	1	*0.2*
*Liner*		
UHMWPE	255	*38.5*
XLPE	407	*61.5*
*Heads*		
Biolox Forte 28	173	*26.2*
Biolox Forte 32	82	*12.4*
Biolox Delta 28	171	*25.9*
Biolox Delta 32	157	*23.6*
Biolox Delta 36	60	*9.0*
Metal 32	19	*2.9*

**Table 2:** Stem pairing

Stem	*N*	%
CFP	430	64.9
CBC	49	7.4
CLS	23	3.5
LC	15	2.3
SP2	118	17.8
SPS	20	3.1
Stellaris	6	0.9
Wagner	1	0.1

(CFP and SP2, Waldemar Link, Hamburg, Germany; CBC and Stellaris, Mathys ltd, Bettlach, Switzerland; CLS and Wagner, Zimmer Inc., Warsaw, IN, USA; SPS, Symbios Orthopaedics, Exeter, UK; LC, Samo SPA, Bologna, Italy)

**Table 3:** Smoking status and relevant comorbidities

Smoking status	*N*	%
Current	110	*23.8*
Former	13	*2.9*

Comorbidities	*N*	*%*
Diabetes	43	*6.5*
Autoimmune diseases	32	*4.8*
Chronic kidney disease	24	*3.6*
Hepatopathy	11	*1.7*
Sickle cell disease	8	*1.2*
Mild cerebral palsy	2	*0.3*

### Clinical and radiographic results

HHS at the end of FU was 87.1 ± 13.8, VAS 1.2 ± 1.1, OHS 41.3 ± 5.4. HHS results proved excellent in 237 subjects (51.6%), good in 160 (34.7%), fair in 26 (5.6%) and poor in 37 (8.1%): OHS outcomes were satisfactory for 307 patients (66.7%), mild in 126 (27.5%), fair in 21 (4.5%) and poor in 6 (1.3%). In 14 cases (3.6%), the patients reported persistent thigh pain. No cases of psoas or femoral nerve impingement were observed.

Osteolysis on the acetabular side was observed in 53 (10.1%) hips, concerning zone I in 26 cases (4.9%), zone II in 94 (17.9%), 19 in zone III (3.6%): in some cases, it involved more than one area in the same hip. Mean acetabular inclination was 46.5° ± 4.3°. On the femur, osteolysis was more frequent in Gruen zone I (34.9% of the cases), zone II (17.7%) and zone VII (23.6%). Mean LDD was 1,45 mm, with a SD of 1.93 mm, and NRR, evaluated in the sub-cohort of patients treated with neck sparing stem, 0.26 ± 0.28. HO were present in 176 (33.6%) cases, more frequently classifiable as Brooker I (70 hips) or II (67).

### Complications

Sixty-six (12.5%) patients underwent revision surgery for any reason. Among them 50 had developed aseptic loosening (AL) of the cup (13, 2.4%), of the stem (11, 2.1%) or both (26, 4.9%). Periprosthetic joint infection occurred in 8 patients (1.6%), and 7 (1.3%) cases were treated with a two-stage revision procedure: in one patient, a suppressive therapy was preferred due to critical clinical conditions. Every case of PJI was confirmed with a preoperative arthrocentesis.

Other causes of cup revision include polyethylene wear (2 cases, 0.4%) and recurrent dislocation (2, 0.4%). Polyethylene wear occurred in 17 patients globally, but in 15 of them, liner substitution was sufficient to restore a good hip function: 2 cases of recurrent dislocation were treated conservatively. Complications are resumed in Table 4.

**Table 4:** Complications

	*N*	%
*Complications*		
Revisions	66	*12.5*
Aseptic loosening cup	13	*2.4*
Aseptic loosening stem	11	*2.1*
Aseptic loosening cup + stem	26	*4.9*
Periprosthetic joint infection	7	*1.3*
Polyethylene wear	2	*0.4*
Recurrent dislocation	2	*0.4*
Periprosthetic fracture	5	*0.9*
*Reoperations*		
Ceramic rupture	1	*0.2*
Polyethylene wear (liner substitution)	15	*2.9*
Recurrent dislocation (conservative)	2	*0.4*
Periprosthetic joint infection (suppressive)	1	*0.2*

Acetabular bone loss was classified according to Paprosky classification [12]. In every case, cup revision was performed using a Trilogy Trabecular Metal cup (Zimmer Inc., Warsaw, IN, USA).

**Fig. 1:**
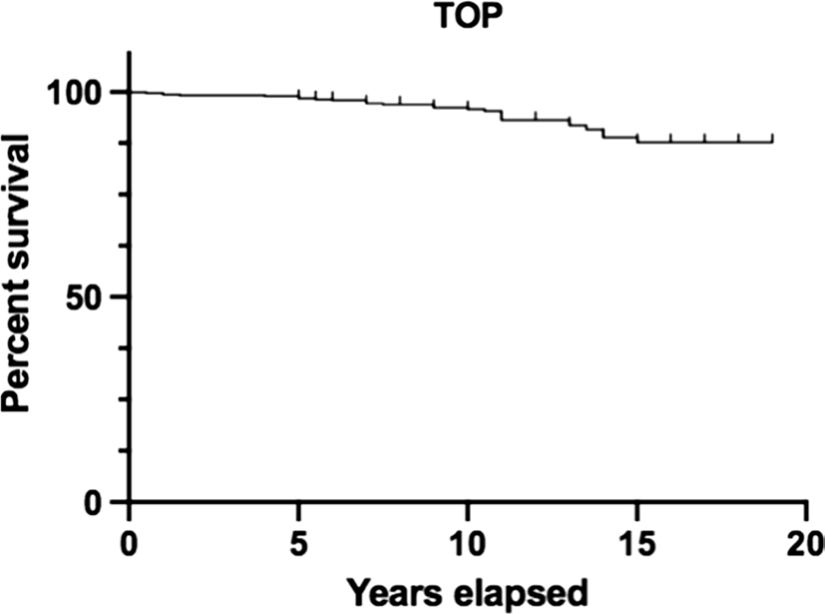
Kaplan–Meier curve of TOP cup survival

**Fig. 2:**
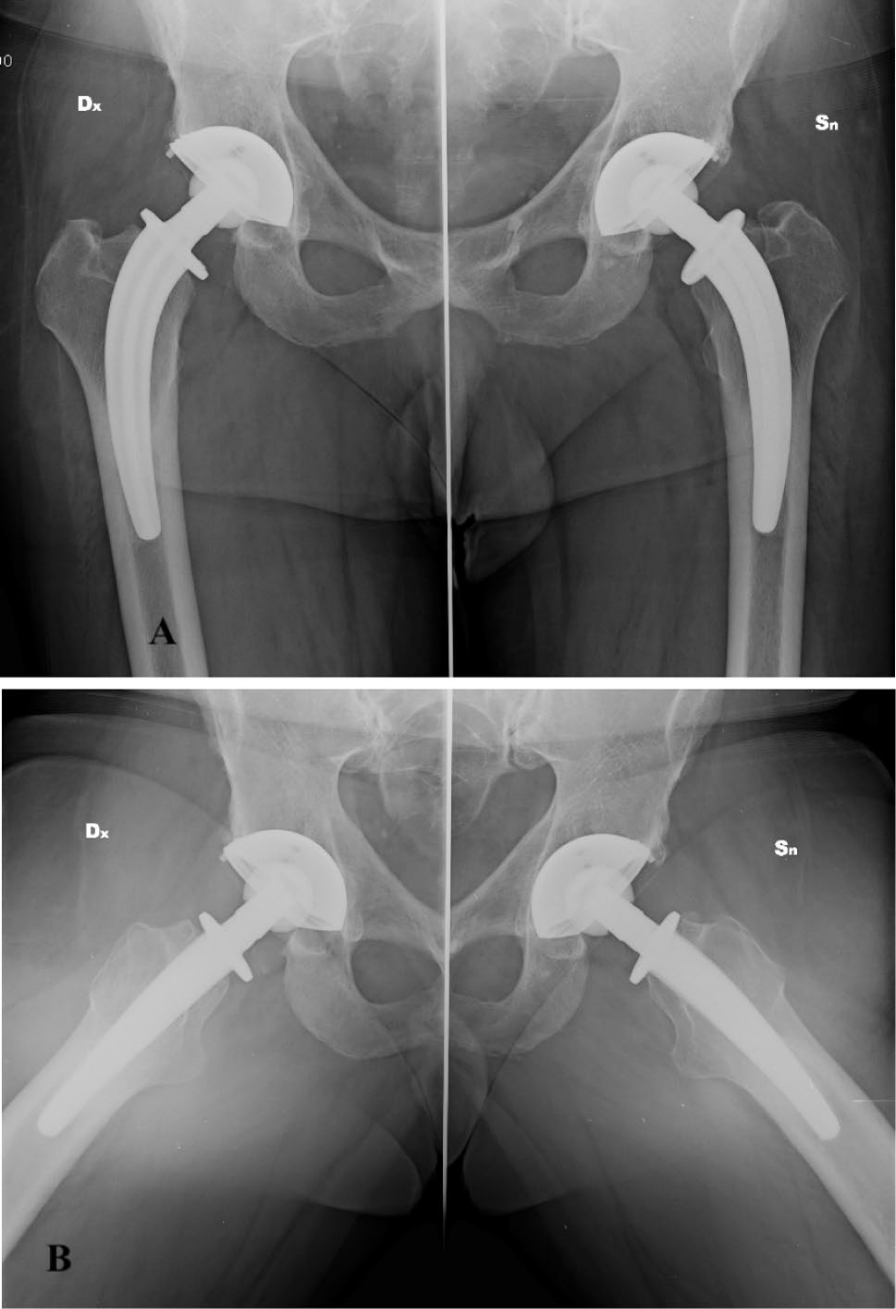
12 years FU in a bilateral TOP cup, paired with CFP stem (Waldemar Link, Hamburg, Germany).**a** AP view. **b** Axial view of the stem. On the right side, a slight reabsorption of the femoral neck (NRR = 0.18)

Globally, in our cohort, there was a cup revision rate of 9.5% (50 cases), with a 90.5% survival rate at the end of FU. Mean cup revision time was 10.1 ± 4.3 years. Figures 1 and 2 shows the Kaplan–Meier curve indicating cup survival.

Other reasons for revision surgery were stem loosening (11 patients) and periprosthetic fracture (5).

One patient suffered of ceramic head rupture and required one further surgical procedure.

There was no correlation (*p* = 0.09) between complication insurgence and patients’ comorbidities: a significant correlation (*p* = 0.001) was found between HHS results and osteolysis, demonstrating a tight correlation between clinical and radiological results.

## Discussion

TOP cup showed a good survivorship at a long-term FU (90.5%). As far as we know, this is one of the few reports on a long-term FU [range 5–21 years] for this type of cup. The use of TOP cup has been previously validated in short-term clinical studies [13–16], and with analysis performed on bone reabsorption [17]. Some authors advocated an increased risk of revision in cups coated with hydroxyapatite [18, 19]. The difference we report in terms of cup survival could be related to the fact that the TOP cup relies on a different principle than the cups examined by Lazarinis and coll. (partially treated, slight HA coating, partial hemispheric profile). As highlighted by the authors, in the population they examined differences were seen among the various types of cups examined: furthermore, the use of femoral heads of smaller sizes and different tribology (metal heads) could make the two cohorts of patients dissimilar. Main reason for cup revision was AL (isolated or combined with stem loosening, 13 and 26 cases, respectively): comparing the results reported by Wacha et al. [20], we observed a higher rate of AL, but a lower rate of dislocations and PJI: a longer FU in our group of patients could be the explanation for a significant difference in cup survival due to AL. Figure 2 shows the radiographic 12 years FU in a patient with bilateral TOP cup paired with bilateral CFP stem.

In the majority of cases, the cup was paired with a CFP stem, in accordance with the principles of TSS: using a neck sparing stem such as the CFP allows the recreation of an artificial joint similar to the native hip, especially in terms of load transmission. This feature is important to prevent the stress shielding effect that could lead to implant mobilization [3].

Clinical evaluation showed excellent or good outcomes in the majority of patients (HHS, 86.3% and OHS, 94,2%), as previously described in shorter term FU studies [14, 21].

For every patient, an accurate preoperative planning was performed, in order to select the most suitable cup/stem pairing considering preoperative diagnosis, bone morphology and risk factors.

As previously indicated from Lazarinis et al. [22], TOP provides good stability, though proximal cancellous bone reabsorption is present in the early radiological evaluation after positioning. The significant correlation found between clinical and radiological outcomes, demonstrates how osteolysis in an active process in the operated hip and cannot be underestimated. The reduction in cancellous bone in Charnley and DeLee zones I and II is believed to be related to some sort of stress shielding: Gruen zones I and VII are the most affected on femoral side, accounting in combination for the 58.5% of all the osteolysis observed in the cohort, and it is current opinion that this pattern is referable to load transmission from the stem to the more distal part of the femur [10, 15, 20, 22, 23]. There were no records of psoas or femoral nerve impingement: the incidence of this complication using other cups is as high as 4.3% and 2.4%, respectively [24, 25], and it is caused more often by the anteroinferior rim of the acetabulum. This complication could determine the necessity of a reoperation (e.g., cup reorientation or iliopsoas tendon tenotomy) [24]: the medio-ventral recess in the TOP cup avoids tendon and nerve irritation during hip flexion.

TOP cup permits an anatomical positioning even in complex acetabular, in which a more vertical implantation is required, achieving an adequate coverage thanks to its biequatorial feature [26, 27]. This is confirmed by the relatively low rate of dislocations (0.7%): in the literature, the incidence of dislocation following THA varies between 0.2% and 10% [28].

This study presents several limitations; first of all, its retrospective nature does not allow a control-case confrontation, in order to assess, for instance, differences in the use of other implants or different surgical techniques. Surgeries were performed by three different surgeons during a vast period of time; therefore, there could be biases on the final outcomes. Furthermore, the drop-out rate of 21,8% could not be ignored. Despite these limitations, the single-centre experience, long-term follow-up and the conspicuous number of patients are an undeniable strength: additionally, even though the procedures were performed by three different operators, surgical technique (i.e., surgical approach, component positioning) and pre- and postoperative care were the same for all the patients.

## Conclusions

Uncemented press-fit cup is the preferred choice in contemporary hip replacement. Different designs are now available on the market. The TOP cup is a modern and low profile cup, which provides good clinical outcomes and reliability even for young patient over a long period of time. A precise preoperative planning and surgical technique are mandatory to achieve cup stability and lower revision rates. Further studies with similar FU on different cohorts of patients are necessary to confirm these results.
